# The Role of Emotional Intelligence in Preventing Suicidal Behaviors: A Systematic Review and Meta-analysis

**DOI:** 10.34172/jrhs.2025.178

**Published:** 2025-04-01

**Authors:** Nahid Darvishi, Mehran Farhadi, Jalal Poorolajal

**Affiliations:** ^1^Department of Psychology, Sanandaj Branch, Islamic Azad University, Sanandaj, Iran; ^2^Department of Psychology, Faculty of Economics and Social Sciences, Bu-Ali Sina University Hamadan, Iran; ^3^Department of Epidemiology, School of Public Health, Hamadan University of Medical Sciences, Hamadan, Iran; ^4^Modeling of Noncommunicable Diseases Research Center, Hamadan University of Medical Sciences, Hamadan, Iran

**Keywords:** Emotional intelligence, Suicidal ideation, Suicide attempts, Meta-analysis

## Abstract

**Background:** Despite extensive research examining the relationship between emotional intelligence (EI) and suicide, the extent to which EI can prevent suicidal behaviors remains unclear. This systematic review and meta-analysis aimed to investigate this relationship.

**Study Design:** This is a systematic review and meta-analysis study.

**Methods:** A comprehensive search was conducted on PubMed, Web of Science, and Scopus databases up to September 8, 2024. Studies exploring the relationship between mean EI scores and suicidal behaviors were included. Between-study heterogeneity was assessed using I^2^ statistics, and the likelihood of publication bias was evaluated using Begg’s and Egger’s tests. The primary outcome was the standardized mean difference (SMD) in EI scores between individuals with and without suicidal behaviors or ideation using a random-effects model.

**Results:** Out of 3470 studies initially identified, 10 studies (including 2532 participants) met the eligibility criteria. The results showed that individuals without suicidal attempts had significantly higher overall EI scores than those who did (SMD=0.99; 95% CI: 0.58-1.40; *P*<0.001). Similarly, individuals without suicidal ideation had significantly higher EI scores (SMD=0.47; 95% CI: 0.13-0.82; *P*=0.007). No evidence of publication bias was found (*P*=0.525).

**Conclusion:** These findings suggest that higher EI is associated with a reduced risk of suicidal ideation and attempts. Incorporating EI into psychological treatments and suicide prevention programs may be beneficial in mitigating suicidal behaviors.

## Background

 Suicide is a leading global cause of death, with one person taking their own life every 40 seconds worldwide.^1^ Each year, over 800 000 individuals die by suicide worldwide, and for every suicide death, approximately 20 suicide attempts occur.^1^ It is a pressing issue that demands effective prevention strategies. Recognizing the importance of promoting mental health and overall well-being, a global target has been set to reduce premature mortality by one-third by 2030, encompassing efforts to address suicide^2^. Protective factors such as social support, problem-solving skills, strong personal relationships, positive coping techniques, and personal belief systems have all been identified as crucial elements in suicide prevention.^1,3,4^

 One promising technique for reducing suicide rates is the development of emotional intelligence (EI). EI refers to the ability to identify and regulate one’s own emotions, as well as to understand and influence the emotions of others.^5^ Certain components of EI have been found to protect against psychological stress and contribute to strong social support, which, in turn, can help prevent depression and suicidal thoughts.^6^

 Research examining the link between EL and suicide has consistently reported that individuals who attempt suicide tend to have lower levels of EI.^7-16^ A systematic review conducted by Domínguez-García and Fernández-Berrocal in 2018, covering both English and Spanish literature, specifically highlighted the significant role of high levels of EI in preventing suicidal behavior.^17^

 While existing research has established a clear connection between EI and suicide, an important gap remains that needs to be addressed. Specifically, no meta-analysis has been conducted to quantitatively assess EI’s potential for preventing suicidal behaviors. This lack of comprehensive analysis limits our understanding of the potential of EI as a prevention strategy. Therefore, this study aimed to fill this gap by providing an up-to-date and thorough analysis of the latest scientific evidence on the relationship between EI and suicide prevention. Through this analysis, we hope to shed light on the potential of EI as a promising strategy for reducing the incidence of suicide.

## Methods

###  Eligibility criteria

 Eligibility criteria were established using the Population, Intervention/Exposure, Comparison, Outcome, and Study Design (PICOS) framework to ensure a comprehensive and systematic approach to selecting studies for this review.^18^ This framework clearly defines the key components involved in examining the relationship between EI and suicidal behaviors.

*Population*: Eligible study populations included individuals from the general population, including community-dwelling individuals, students, individuals with alcohol dependence, and patients with mental illness.

*Exposure*: The primary exposure of interest was the average EI score, measured using any validated tool or questionnaire. Studies were included if they reported EI scores quantitatively such as mean and standard deviation (SD). Higher EI scores were compared with lower EI scores.

*Outcome*: The primary outcomes of interest encompassed all types of suicidal behaviors, including suicidal ideation, suicide planning, suicide attempts, and death by suicide.

*Study design*: Observational studies, specifically case-control studies and cross-sectional studies, that assess the relationship between EI and suicidal behaviors were included. There was no restriction based on publication date or language.

###  Search methods 

 A systematic search was conducted across the PubMed, Web of Science, and Scopus databases up to September 8, 2024, to identify relevant studies. Additionally, reference lists of all retrieved studies and pertinent reviews were screened for additional sources. The search terms used were “emotional intelligence” OR “emotional quotient” OR “social intelligence “AND “suicide” OR “suicidal” OR “suicidality”.

###  Study selection

 The search results obtained from electronic databases were combined, and duplicates were removed using the EndNote software. Two authors independently reviewed the titles and abstracts to determine eligibility criteria for inclusion in this review. The authors discussed and resolved disagreements. For further evaluation, the full texts of relevant studies were downloaded.

###  Data extraction 

 Data were extracted from relevant studies and entered into an electronic data sheet created using Stata software. The following information was collected: first author’s name, year of publication, country, language, mean age, gender, study population (general population, students, individuals with mental illness), study design (cross-sectional, case-control), suicidal behaviors (suicidal ideation, suicide attempts), period of suicidal behavior (past month, past year, lifetime), sample size, mean score of EI, and its SD.

###  Methodological quality

 The quality of the studies was assessed using the Newcastle-Ottawa Scale (NOS).^19^ Each study was awarded a maximum of nine stars based on criteria related to selection, comparability, and outcome. Studies with seven or more stars were considered high-quality, while those with fewer than seven stars were considered low-quality.^20^

###  Heterogeneity and publication bias

 Heterogeneity among studies was assessed using the chi-square (χ^2^) test, tau-squared (τ^2^) test,^18^ and I^2^ statistic.^21^ Heterogeneity was categorized as low ( ≤ 50%), moderate (50%-74%), or high ( ≥ 75%), depending on the I^2^ value.^21^ To evaluate potential publication bias, Egger^22^ and Begg^23^ tests were conducted.

###  Data synthesis

 Data were analyzed using Stata software version 14 (StataCorp, College Station, TX, USA) and Review Manager 5. Meta-analysis was performed using a random-effects model at a significance level set at 0.05.^18^ Since different questionnaires and tools have been used to measure EI in various studies, the standardized mean difference (SMD) was used as the summary statistic, along with 95% confidence intervals (CIs), instead of the normal mean difference. SMD values of 0.2, 0.5, and 0.8 were considered small, moderate, and large effects, respectively.^24^

## Results

###  Description of studies

**Figure 1:**
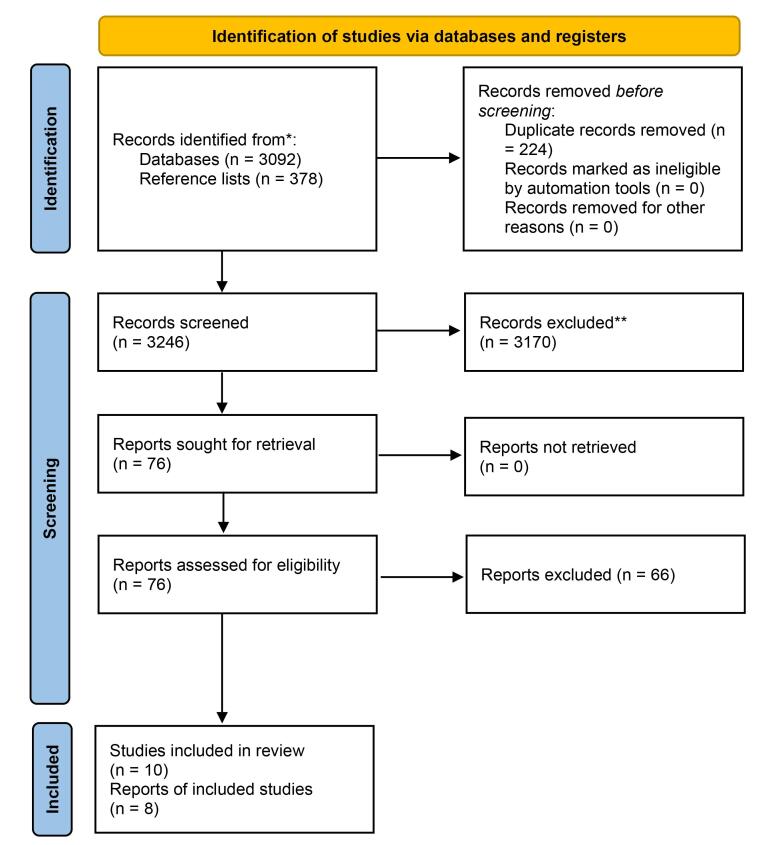
The flow of information through the different phases of the systematic review, including the number of studies identified, screened, and included in the analysis

 A total of 3470 studies were identified, with 3092 studies retrieved from electronic database searches up to September 8, 2024, and an additional 378 articles found through reference list screening. After removing duplicates and ineligible studies, 10 studies involving 2532 individuals were included.^7-16^ However, two studies were excluded from the meta-analysis due to the lack of reported standard deviations for their means (Figure 1). The characteristics of the studies included in this analysis are summarized in Table 1.

**Table 1:** Characteristics of the Included Studies

**First Author**	**Country**	**Language**	**Population**	**Age means (y)**	**Sex**	**Study design**	**Questionnaire**	**Suicide type**	**Suicide time**	**Sample**	**Quality**
Abdollahi^7^	Iran	English	Mental	16.28	Both	Cross-sectional	Schutte	Attempt	Lifetime	202	*****
Barekatain^8^	Iran	Persian	General	26.75	Female	Case-control	Bar-On	Attempt	Past month	160	******
Gómez- Romero^9^	Spain	Spanish	Students	17.94	Both	Cross-sectional	TMMS	Ideation	Lifetime	144	*****
Kopera^10^	Poland	English	Alcoholic	44.64	Both	Cross-sectional	Schutte	Attempt	Lifetime	80	*****
Korkmaz^11^	Turkey	English	General	35.32	Both	Case-control	EIS	Attempt	Past month	50	*******
Moayedi^12^	Iran	English	General	22.49	Both	Case-control	Bar-On	Attempt	Past month	100	******
Motahari^13^	Iran	English	General	Not reported	Both	Case-control	Bar-On	Attempt	Past month	60	*****
Okasha^14^	Egypt	English	Mental	3.70	Both	Cross-sectional	TMMS	Attempt	Lifetime	45	*****
Sojer^15^	Austria	English	Students	24.30	Both	Cross-sectional	SEAS	Ideation	Lifetime	277	******
Tabares^16^	Colombia	Spanish	Students	20.49	Both	Cross-sectional	TMMS	Ideation	Past month	1414	******

###  Synthesis of results

**Figure 2:**
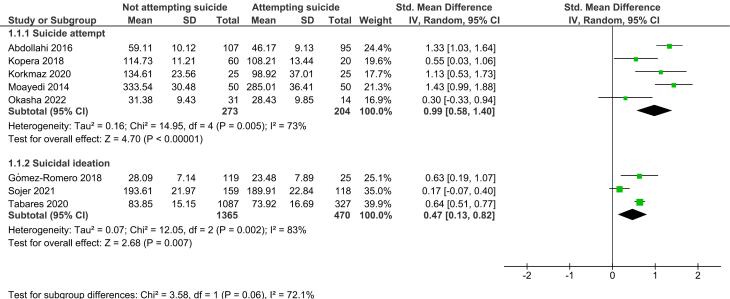
Forest plot of the association between emotional intelligence and suicidal ideation and suicide attempts. *Note.* SMD: Standardized mean difference; The plot shows the SMD and corresponding confidence intervals for each study, as well as the combined SMD for all studies. The figure also includes measures of heterogeneity between studies and statistical tests for publication bias

 The analysis of five studies (Figure 2) indicated that individuals who did not attempt suicide had a significantly higher total EI score compared to those who attempted suicide (SMD = 0.99, 95% CI: 0.58-1.40, *P* < 0.001). This indicates that the mean EI score for individuals who did not attempt suicide was 0.99 points higher than for those who did, representing a large difference. The between-study heterogeneity was moderate (I^2^ = 73%), and the Begg test (*P* = 1.000) and Egger test (*P* = 0.525) showed no evidence of publication bias.

 In addition, as illustrated in Figure 2, the analysis of three studies revealed that individuals without suicidal ideation had significantly higher total EI scores than those with suicidal ideation (SMD = 0.47, 95% CI: 0.13-0.82, *P* = 0.007). This suggests that the overall mean EI score of individuals without suicidal attempts was 0.47 higher than those with suicidal attempts, indicating a moderate difference. Moreover, the between-study heterogeneity in this sub-group was high (I^2^ = 83%).

 Motahari and Rahgozar conducted a case-control study which found significantly higher average EI scores among individuals who did not attempt suicide compared to those who did (354.16 vs. 230.10, *P* < 0.001). However, this study was not included in the meta-analysis because the SDs of the means were not reported.^13^ Similarly, a case-control study by Barekatain et al reported that the mean EI score was significantly higher in individuals who did not attempt suicide than in those who did (356.14 vs. 154.12).^8^ These findings align with the meta-analysis results, suggesting that EI may serve as a protective factor against suicide attempts.

###  Subgroup analysis

Table 2 presents the relationship between EI and suicide attempts across various study designs and population characteristics. Both case-control studies (SMD = 1.33, *P* < 0.001) and cross-sectional studies (SMD = 0.77, *P* < 0.030) indicated that individuals without suicide attempts had significantly higher total EI scores compared to those who did. Additionally, subgroup analysis revealed that, in the general population, individuals who did not attempt suicide had a significantly higher total EI score (SMD = 1.33, *P* < 0.001). In contrast, the association was not significant in populations with mental illness (SMD = 0.86, *P* = 0.100), suggesting that EI may not provide the same protective effect in this group. A study involving alcoholics indicated a modest significant association (SMD = 0.55, *P* = 0.040).

**Table 2:** Meta-analysis of the association between emotional intelligence and suicide attempts: subgroup analysis by study design and population characteristics

**Subgroups**	**No. of Studies**	**Suicidal Behavior**	**I**^2^	**SMD (95% CI)**	***P*** ** value**
Study design					
Case-control	2	Attempted suicide	0.0%	1.33 (0.97, 1.68)	0.001
Cross-sectional	3	Attempted suicide	84%	0.77 (0.09, 1.44)	0.030
Study population					
General	2	Attempted suicide	0.0%	1.33 (0.97, 1.68)	0.001
Mental illness	2	Attempted suicide	88%	0.86 (-0.15, 1.86)	0.100
Alcoholics	1	Attempted suicide	-	0.55 (0.03, 1.06)	0.040

*Note.* SMD: Standardized mean difference; CI: Confidence interval.

## Discussion

 The present meta-analysis aimed to investigate the relationship between EI and suicidal behaviors. The results showed that individuals without suicidal ideations and behaviors had significantly higher total EI scores compared to those who attempted suicide. This finding suggests that higher levels of EI may serve as a protective factor against suicidal behaviors. However, the moderate heterogeneity observed between studies indicates that differences in study design, population characteristics, and other factors could contribute to the observed variation in the results.

 EI refers to the ability to perceive, use, understand, manage, and regulate emotions effectively. Individuals with high EI can recognize and label their own emotions and can understand other individuals’ emotions. They can use emotional information to guide their thoughts and behaviors, distinguish between different feelings, and appropriately label them. Additionally, they can regulate their own emotions, adapting effectively to various situations. High EI is associated with better emotional regulation, effective communication, and the ability to form strong relationships with others.^25^

 Research has demonstrated that individuals with high EI tend to experience better mental health and social well-being. EI can significantly impact how we think, feel, and act, influencing our ability to manage stress, interact effectively with others, and make sound decisions. Specifically, individuals with high EI are better able to regulate their emotions and respond to the emotions of others in a positive and adaptive manner. This can lead to more fulfilling relationships, greater resilience in the face of adversity, and improved overall well-being.^26^

 EI is closely linked to empathy, as it enables individuals to connect their own emotional experiences with those of others. Individuals with high EI tend to be more empathetic, are better capable of regulating their emotions, and respond appropriately to the emotions of others. In contrast, people with low EI may experience emotional emptiness and exhibit inappropriate emotional reactions. As a result, low EI can be considered a risk factor for developing mental illnesses and can create difficulties in adapting to one’s environment. Therefore, developing EI skills plays a crucial role in improving mental health outcomes and enhancing overall well-being.^27^

 Individuals with low EI may struggle with effective problem-solving and adaptive coping skills when faced with psychological stressors. This can reduce the ability to regulate emotions and respond appropriately to challenging situations.^28^

 Certain components of EI can serve as protective factors against psychological stress. Individuals skilled in emotional regulation tend to experience higher levels of social support, which can help mitigate depression and suicidal thoughts.^6^ EI has been found to make a distinct contribution to understanding the relationship between stress and mental health outcomes such as depression, anxiety, and suicidal thoughts.^29^

 Our results suggest that EI may have a protective effect against suicide attempts. However, suicide is a complex phenomenon that involves multiple interacting risks and protective factors, which cannot be reduced to discrete components.^30-33^ Instead, these factors must be considered holistically. While risk factors increase the likelihood of suicide, protective factors can help reduce it. When risk and protective factors are balanced, or when protective factors outweigh risk factors, suicide may be prevented.^34^ Therefore, the role of EI in suicide prevention should be considered alongside other influential factors. Developing and strengthening protective factors, including EI, may serve as an important strategy for suicide prevention and overall mental health and well-being promotion.

 There are several limitations to this meta-analysis that should be noted. First, the number of eligible studies investigating the relationship between EI and suicidal ideation and attempts was limited. Therefore, additional research is needed to establish a more robust association between EI and different forms of suicidal behaviors. Second, the studies included in this review used different tools to measure EI, which could have affected the comparability of the findings. To address this, we used SMD to combine and report the results across studies. Third, although the Begg and Egger tests were employed to assess publication bias, it is important to note that these tests were applied to only eight studies included in the meta-analysis, which is below the recommended threshold of ten studies for reliable results. Consequently, the statistical power of these tests may be limited, potentially affecting the robustness of our findings regarding publication bias. Finally, the high heterogeneity among the included studies was another limitation of this review. Despite conducting a subgroup analysis based on study design and population characteristics, homogeneity was only established for the subgroup of case-control studies and the general population. The remaining subgroups exhibited significant heterogeneity, which may impact the overall interpretation of the results. Nevertheless, this meta-analysis provides valuable insights into the potential role of EI as a protective factor against suicidal behaviors and highlights the need for further research in this area.

 Based on these findings, health policy recommendations could include the integration of EI training programs into suicide prevention strategies. These programs could be offered to individuals who are at a higher risk for suicide such as those with a history of mental illness or those experiencing significant life stressors. Additionally, mental health professionals could integrate EI assessments into their clinical practice to identify individuals who may benefit from targeted EI interventions. Further research is needed to determine the most effective ways to incorporate EI training and assessment into suicide prevention efforts. However, the findings of this meta-analysis suggest that enhancing EI may be a promising strategy for reducing suicide risk.

**Highlights**

- Individuals who did not attempt suicide had a significantly higher total score of emotional intelligence (EI) compared to those who attempted suicide.
- The overall standard mean score of EI in individuals who did not attempt suicide was 0.93 higher than those who attempted suicide, indicating that EI may be a protective factor against suicide attempts.
- Although the relationship between EI and suicidal ideation was not statistically significant, further research is needed to explore this relationship in more detail.

## Conclusion

 The results of this meta-analysis suggest that EI may serve as a protective factor against suicidal thoughts and behaviors. Individuals with higher levels of EI may possess better emotional regulation skills, social support, and coping mechanisms, ultimately reducing their susceptibility to suicide. However, these findings should be interpreted cautiously due to the moderate variability between studies and the limited number of studies exploring the connection between EI and suicide. Further research is necessary to gain a more comprehensive understanding of the potential impact of EI in preventing suicidal behaviors.

## Acknowledgments

 The results were derived as part of a Ph. D. thesis in Psychology. The authors would like to acknowledge the Vice-Chancellor for Research and Technology at Azad University for approving the study.

## Competing Interests

 The authors declare no conflict of interests related to the publication of this study.

## Ethical Approval

 This study is a review article that does not involve human or animal subjects. It provides a comprehensive analysis of the existing literature on the topic.

## Funding

 No financial support was provided for this study.
